# Event and Comment

**Published:** 1917-10

**Authors:** 


### DENTAL REGISTER

Vol. LXXI OCTOBER, 1917 No. 10

### EVENT AND COMMENT

CONSERVING OUR DRUGS.—We are printing under this title in this issue an appeal to all who use drugs to use all possible care in saving of drugs and chemicals. If this war does no other good it certainly ought to teach us the value of saving our resources as we have never before thought it necessary to do. The drugs used by the dentist are not large in quantity, but most of them are of the expensive sorts, and some are becoming scarce because of their use in the various war necessities. In fact, it is more and more evident that we must economize in every possible direction. Let us do all we can to save, except where such efforts might lead to dangerous treatment of our patients. We shall probably be surprised to find out how much less we can get on with after we have made a thoroughly conscientious effort to save things. It ought to be good for us. So let us do it cheerfully and faithfully.

POST-GRADUATE INSTRUCTION.—Columbia University has announced a course of post-graduate instruction in the technical subjects of dental practice. It is to be administered under the department having charge of extension teaching, rather than by the faculty of the newly-organized dental school. Courses are offered in operative dentistry, plate prosthesis, root canal technic and roentgenology, dental ceramics, periodontia, anesthesia and exodontia. Courses vary in time from two to ten weeks on the different subjects. The instruction is to be given by a number of men practicing as specialists in the various subjects, and the laboratories and clinical facilities of the university will be used for purposes of instruction. There is no doubt that such a course will be of great value in enabling dentists to perfect their technical attainments, and we should think it would be well supported, providing the instruction is adequate. It would do no dentist any harm to get out of his office occasionally and put himself in the care of some one who is doing an unusually good line of work in any department of practice. It should be stimulating and prevent retrogression or bigotry in practice. New York has advantages in the way of clinical material and skillful practitioners, and a school in which advance methods only are taught should be a most helpful and welcome addition to our educational institutions.

CONNECTICUT'S PART IN DENTAL DEVELOPMENT.—We have just read a little pamphlet written by Dr. James McManus, of Hartford, Connecticut, on the careers of some natives of that state who have helped greatly in establishing dentistry as a profession in this country. It certainly should be a matter of pride to know that Horace Hayden, the acknowledged founder of dental education and literature, and the man who more than any other in its formative period laid out the plans on which a new and honored profession should be built, was born in any one's neighborhood. Dr. McManus also rejoices that Dr. Horace Wells, the discoverer of general anesthesia, was a Connecticut citizen. Two of the most illustrious men ever connected with the dental profession. He might have mentioned many other illustrious citizens of his state, some of whom are not yet dead, who have been strong men in making dentistry acknowledged, both at home and abroad, as one of the most benevolent of the scientific professions. Material of this sort should be made and preserved in every state in the country and be accessible to dental students in our colleges. The lives of these men should serve to stimulate others to greater endeavors in the development of professional ideals and better educational standards.

STANDARDIZING DENTAL EDUCATION. Several years ago the Educational Council of the American Medical Association made a systematic examination of all the medical colleges and classified them as to their efficiency as educational institutions. The result was to gradually eliminate some of the schools and to bring all up to a more equal degree of efficiency. It probably is true that some schools were discontinued that deserved to exist, because of the character of the teaching done by them; but the modern medical practitioner needs a broader training than can be had in many unendowed medical schools, and therefore some good schools were forced to quit because they could not supply the facilities for a grade of instruction that was thought necessary by the American Medical Association, and the different state boards of examiners. In the dental field there has been no such examination or effort to force a standard of ideal dental training, except as this has come about, because of much discussion and the action taken two years ago by the dental faculties associations and the enactment by a few states of registration laws which call for a more exacting dental education than had formerly been accepted. The New York Board of Regents, acting under the new law, enacted in that state last year, has seen fit to make an effort to put into effect what may well be called a standard of dental education. The standard adopted demands a four year course of instruction with preliminary education equal to a specified high school course. The dental college curriculum is also definitely specified not only as to subjects taught but the actual hours each subject shall be taught. The New York educational department is not itself a teaching body, but has control of the professional educational institutions of the state of New York to the extent of licensing the output to practice in that state. It therefore has demanded that graduates of all dental colleges who want to register for practice in New York, shall have completed the course of study specified by that body. This action therefore standardizes dental education not only for all New York state dental colleges, but also for all colleges in other states or countries whose graduates wish to locate for practice in New York. It would seem that this is the beginning of a policy which must lead to greater unification of teaching in all our schools, as so many colleges are likely to be compelled to meet this New York requirement, that it will influence the legislation in so many other states, that in a short time all colleges will be teaching practically the New York curriculum. It is a more comprehensive requirement than has been demanded in the majority of our colleges and it will require more and better teaching. There is a possibility that some colleges can not afford the extra cost of its administration and it may be that we shall have an experience similar to the medical colleges. It would be unfortunate to have any of our colleges close their doors at this time, because we need every new graduate we can get to cope with the ever increasing demands for dental service. At the same time it may be wiser to increase the standards of education and thereby secure more efficient practitioners rather than to be satisfied with our present attainments. We must meet in a professional manner the requirements for scientific professional practice that have never before been demanded. If there was a good and sufficient reason for better educated physicians, there is also a greater demand for better trained dentists now than ever before; and as we have no other source of supply than our dental colleges, every effort made to make them more efficient should be gladly welcomed.

